# An mTOR feedback loop mediates the ‘flare’ (‘rebound’) response to MET tyrosine kinase inhibition

**DOI:** 10.1038/s41598-023-28648-3

**Published:** 2023-01-25

**Authors:** D. M. Altintas, M. Cerqua, A. De Laurentiis, L. Trusolino, C. Boccaccio, P. M. Comoglio

**Affiliations:** 1 IFOM ETS - The AIRC Institute of Molecular Oncology, Via Adamello 16, 20139 Milano, Italy; 2 Candiolo Cancer Institute, FPO-IRCCS, Strada Provinciale 142, 10060 Candiolo, TO Italy; 3 Department of Oncology, University of Torino, Strada Provinciale 142, 10060 Candiolo, TO Italy

**Keywords:** Cancer therapy, Oncogenes

## Abstract

Targeted therapy significantly impairs tumour growth but suffers from limitations, among which the ‘flare’ (‘rebound’) effect. Among cancers driven by tyrosine kinase receptors, those relying on alterations of the *MET* oncogene benefit from treatment by specific inhibitors. Previously, we reported that discontinuation of MET tyrosine kinase receptor inhibition causes ‘rebound’ activation of the oncogene, with a post-treatment transient hyperphosphorylation phase that culminates into a dramatic increase in cancer cell proliferation. The molecular mechanisms behind the ‘MET burst’ after treatment cessation are unknown but critically important for patients. Here we identify a positive feedback loop mediated by the AKT/mTOR pathway leading to (a) enhanced MET translation by activating p70S6K and 4EBP1 and (b) MET hyper-phosphorylation by inactivation of the tyrosine-phosphatase PTP1B. The latter effect is due to m-TOR-driven PTP1B phosphorylation of the inhibitory residues *Ser*^50^ and *Ser*^378^. These data provide in vitro evidence for the use of mTOR inhibitors to prevent the ’flare effect’ in MET targeted therapy, with potential applicative ramifications for patient clinical management.

## Introduction

Targeted therapies were a breakthrough in cancer research, proving our ability to translate cutting-edge understanding of cancer cells’ vulnerabilities into the design of mechanism-specific treatment strategies. They significantly improved the disease-free survival time of patients with tumours dependent on the inhibited target (‘oncogene addiction’)^1^. MET, the receptor tyrosine kinase for hepatocyte growth factor, stands among the top five proteins for which targeted therapies have been developed with successful applications in the clinic^2^. Genetic alterations or gene amplification unleash MET-driven invasive growth in a large panel of cancer types^3^. Accordingly, kinase inhibitors or antibodies induced objective responses in patients with (or preclinical models of) MET-addicted tumours, including non-small cell lung cancer (NSCLC), lung squamous carcinoma, gastric cancer, colorectal adenocarcinoma, melanoma, gliomas, and renal cancer^1,3–14^. However, even in cases experiencing exceptional responses, therapeutic resistance inevitably ensues^15–17^. In current clinical practice, the line of treatment is dismissed when resistance arises. In many patients, discontinuation of kinase inhibitors results in rapid tumour regrowth: this phenomenon is known as *disease ‘flare’* or *tumour ’rebound’*^18–21^. Its real incidence is poorly known, and the prognosis is dismal^22^. The biology and molecular mechanisms behind the ‘flare’ (rebound) phenomenon, either cell-autonomous or mediated by the tumour microenvironment, are largely unexplored. We previously found that abrupt termination of MET kinase inhibition unleashes the ‘flare’ effect^23^. In this paper, we provide the mechanistic explanation for this effect by describing a dual regulation of the MET burst: sudden discontinuation of the targeted therapy leads to restored kinase activity followed by the activation of the AKT/mTOR pathway, resulting in (1) enhanced translation of MET and (2) inactivation of the PTP1B phosphotyrosine-phosphatase, one of the major negative regulators of MET^24–26^. Cancer cells adopt this shrewd dual strategy to increase MET total protein quantity and MET phosphorylation (activation), leading to a rapid resumption of cancer cell proliferation. We demonstrate that the ‘flare effect’ is avoided by specific AKT or mTOR inhibitors and thus provide a proof-of-concept foundation for strategies improving MET targeted therapy.

## Results

### Reactivation of the MET/AKT axis drives the ‘flare’ effect

**Figure 1:**
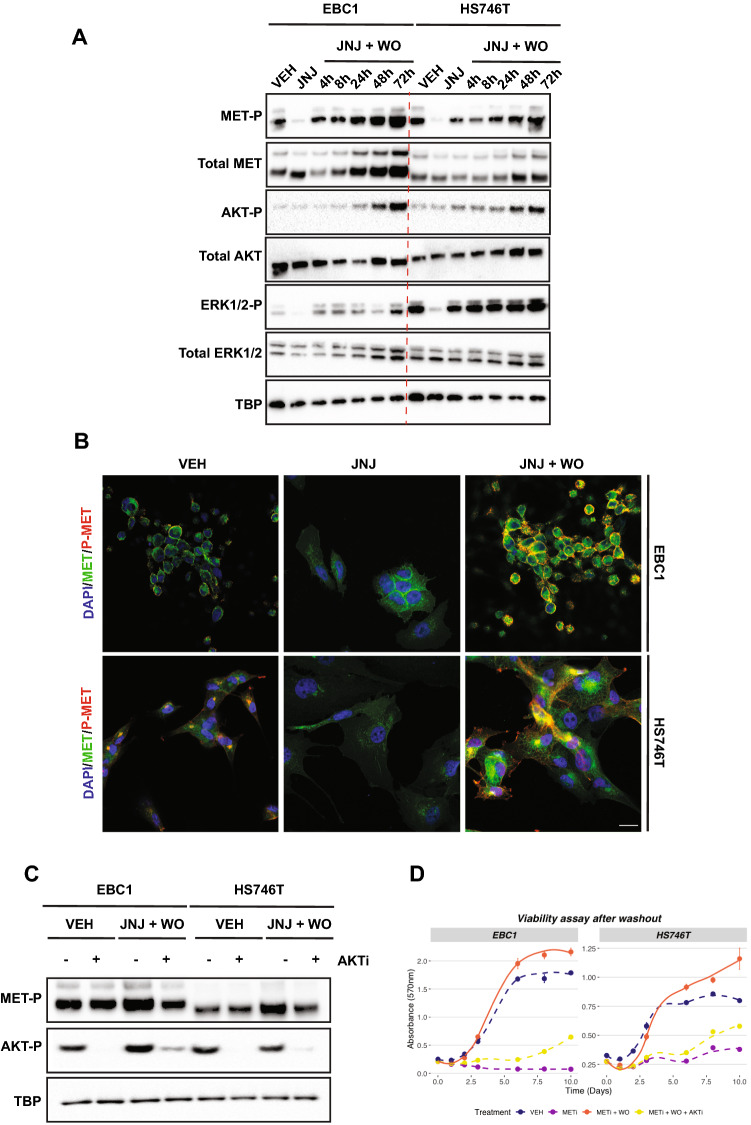
MET/AKT axis drives the ‘flare’ effect. (**A**) EBC1 and HS746T cell lines were treated ON with JNJ-605 or DMSO (VEH). Following inhibitor withdrawal (WO), cells were harvested at the indicated time points (h). Total cellular lysates from the samples shown on top were immunoblotted, as indicated on the left. Black-edged rectangles delineate the blot. The dashed red vertical line highlights the separation between cell lines for clarity. (**B**) Confocal sections of EBC1 and HS746T cells untreated or subjected to JNJ-605 inhibition +/− WO. Cells were then fixed 48 h later and processed for immunofluorescence by staining with anti-phosphorylated MET (P-MET, 647 nm-red pseudocolour) or total MET (MET, 555 nm-green pseudocolour) antibodies and DAPI (blue pseudocolour). (**C**) EBC1 and HS746T cells were treated with DMSO (VEH) or JNJ. After WO, cells were treated with −/+ MK-2206 (AKTi) for 48 h. Black-edged rectangles delineate the blot. (**D**) Viability assay of EBC1 and HS746T cells subjected to indicated treatments. Results are mean +/− SEM with N = 6.

Treatment of cancer cells bearing an amplified, constitutively active MET oncogene with the MET kinase inhibitor JNJ-38877605 (indicated JNJ-605) blocks proliferation in vitro and halts tumour growth in vivo^1,27–30^. However, inhibitor withdrawal causes rapid MET re-phosphorylation at levels higher than those at the steady state, with the subsequent reactivation of downstream pathways and increased proliferation of cancer cells^23^. Yet, the mechanisms of this phenomenon remain obscure. To enlighten what is behind the flare effect, we used two cell lines derived from two cancer cell types (EBC1 isolated from lung squamous carcinoma and HS746T from gastric carcinoma). These cell lines were carefully chosen to recapitulate the most common alterations of *MET* in cancer leading to MET ‘addiction’: gene amplification (EBC1 cells) and exon14 skipping (HS746T cells)^31^. Overnight JNJ-605 treatment abolished MET phosphorylation (Fig. 1A and B). The washout (WO) of the inhibitor from the culture medium restored MET tyrosine activity, reaching -after 48 h- levels of constitutive phosphorylation higher than those displayed by untreated cells at the steady state *(i.e.,* the ‘flare effect’). The activation (phosphorylation) status of known MET transducers, i.e., P-AKT and P-ERK, was analysed. Remarkably, P-AKT levels were significantly enhanced after WO compared with the pre-treatment steady state, whereas no significant change was observed in P-ERK. The concomitant increase of P-MET and P-AKT after WO led us to hypothesise that the MET/AKT axis might be involved in the flare effect. Notably, the specific AKT inhibitor MK-2206 prevented MET hyperphosphorylation after WO (Fig. 1C) and hampered the burst of cell growth resulting from the WO-related ’flare effect’ (Fig. 1D). Importantly, AKT inhibition prevented the reactivation (phosphorylation) of the upstream MET only after JNJ-605 withdrawal, an effect not observed in JNJ-605-untreated cells (Fig. 1C). These results suggest that JNJ-605 washout results in a positive feedback mechanism on MET re-phosphorylation, in which the AKT pathway acts as an intermediate regulator.

### The mTOR pathway enhances MET protein synthesis

**Figure 2:**
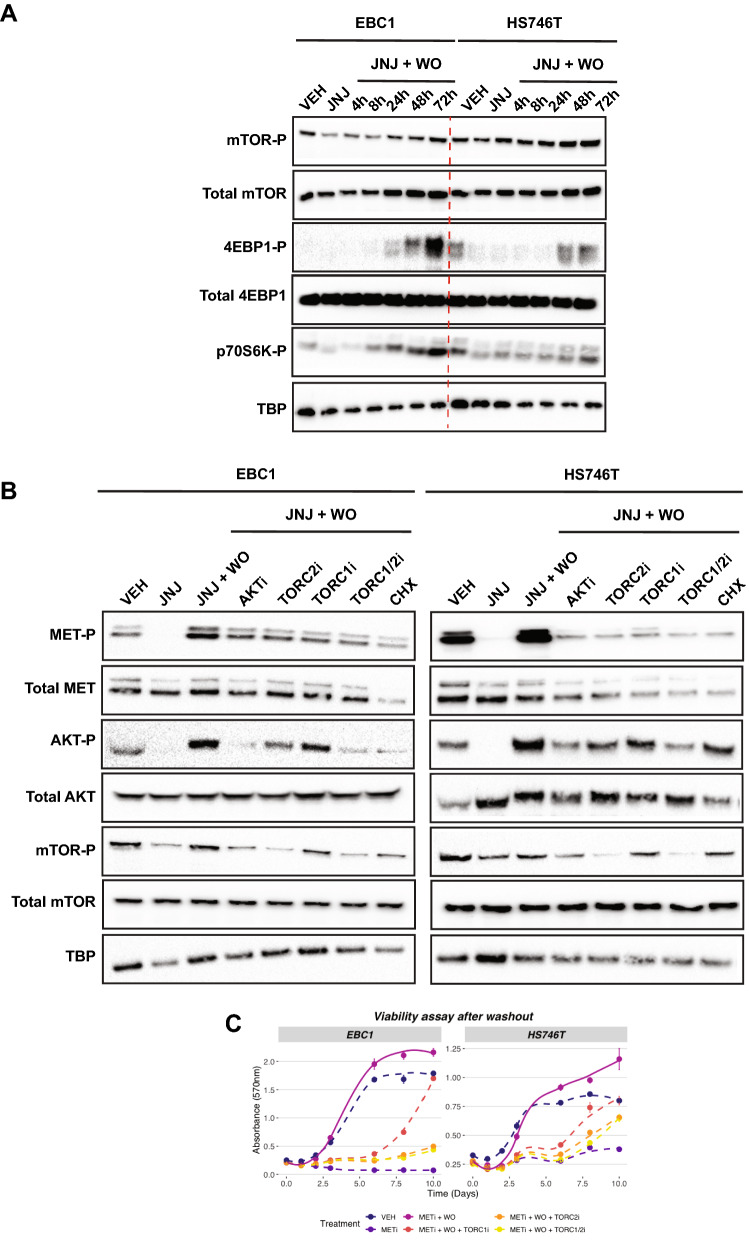
mTOR pathway is activated during the ‘flare’ effect. (**A**) EBC1 and HS746T cell lines were treated ON with JNJ-605 or DMSO (VEH). Following inhibitor withdrawal (WO), cells were harvested at the indicated time points (h). Total cellular lysates from the samples shown on top were immunoblotted, as indicated on the left. Black-edged rectangles delineate the blot. The dashed red vertical line highlights the separation between cell lines for clarity. (**B**) Cells were treated ON with JNJ-605 or DMSO (VEH). After JNJ-605 WO, 1uM of MK-2206 (AKTi), 1uM of JA-AB2-011 (mTORC2i), 0.1uM of rapamycin (mTORC1i), 1uM of Temsirolimus (mTORC1/2i), or 25uM of cycloheximide (CHX) were added in the culture medium. Cells were lysed for immunoblotting 48 h later. Black-edged rectangles delineate the blot. (**C**) Viability assay of EBC1 and HS746T cells subjected to indicated treatments. Results are mean +/− SEM with N = 6.

Interestingly, the kinetics in Fig. 1A showed that the P-MET burst was observed after 48–72 h, a time too long to be fully explained with kinase reactivation after drug discontinuation. We noticed that after WO, total MET protein levels increased. These observations led us to verify the activation status of mTOR, the master regulator of de novo protein synthesis, and the direct target of AKT kinase^32–34^. mTOR phosphorylation levels were gradually increased after MET kinase reactivation following JNJ-605 inhibitor WO in both EBC1 and HS746T cells (Fig. 2A). Accordingly, the downstream protein synthesis effectors in the mTOR pathway, p70S6K and 4EBP1, were phosphorylated (Fig. 2B). Furthermore, the translation inhibitor cycloheximide (CHX) hampered post-treatment MET protein overexpression and hyper-phosphorylation, suggesting that the de novo MET synthesis is critical for the ‘flare’ effect (Fig. 2B).

### The mTOR pathway enhances MET re-phosphorylation

mTOR is part of two structurally and functionally distinct complexes: mTORC1, sensitive to rapamycin, and mTORC2, insensitive to the latter but sensitive to the inhibitor JA/AB2/011^35,36^. mTORC1 is activated by AKT, whereas mTORC2 is directly triggered by PI3K and -in turn- activates AKT^36^. We thus tested the role of each complex on the ’flare effect’ by treating cells with the corresponding specific inhibitors. Treatment of EBC1 or HS746T cells with either inhibitor after JNJ-605 WO blocked the ’flare effect’ (Fig. 2B–C), emphasising that both complexes mediate MET reactivation. The reactivation was also inhibited by the mTOR inhibitor Temsirolimus (Fig. 2B–C).

### mTOR inactivates PTP1B by phosphorylation of Ser^50^ and Ser^378^

**Figure 3:**
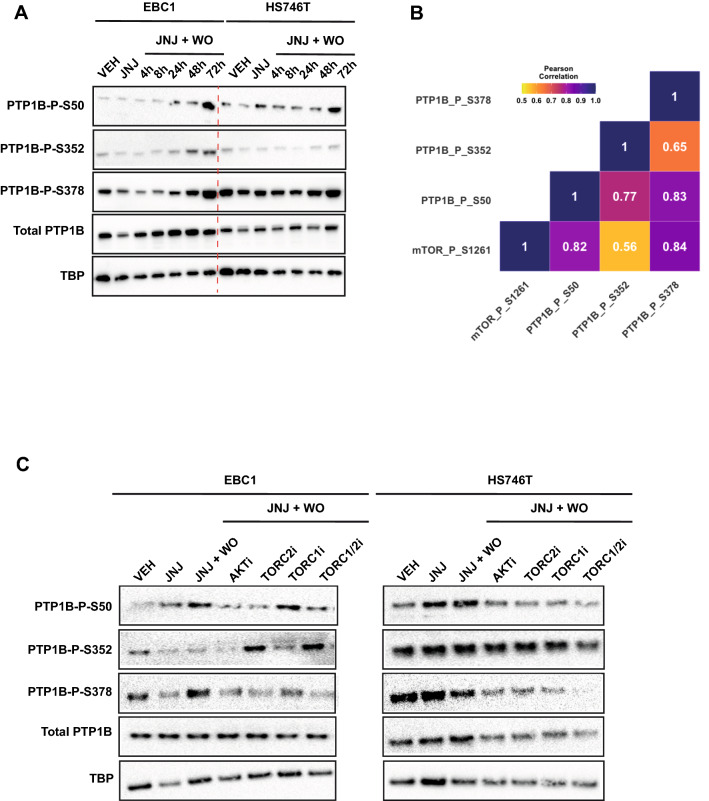
AKT/MTOR axis inactivates PTP1B and drives the flare effect. (**A**) EBC1 and HS746T cell lines were treated ON with JNJ-605 or DMSO (VEH). Following inhibitor withdrawal (WO), cells were harvested at the indicated time points (h). Total cellular lysates from the samples shown on top were immunoblotted, as indicated on the left. Black-edged rectangles delineate the blot. The dashed red vertical line highlights the separation between cell lines for clarity. (**B**) Correlation heatmap showing the relationship between mTOR activation and PTP1B phosphorylation in cancer patients (TCGA data, N = 42 patients). The colour code represents the Pearson correlation score. (**C**) Cells were treated ON with JNJ-605 or DMSO (VEH). After JNJ-605 WO, 1uM of MK-2206 (AKTi), 1uM of JA-AB2-011 (mTORC2i), 0.1uM of rapamycin (mTORC1i), or 1uM of Temsirolimus (mTORC1/2i) were added in the culture medium. Cells were lysed for immunoblotting 48 h later. Black-edged rectangles delineate the blot.

Previously, we noticed that during the ‘flare’ effect, PTP1B levels were affected^23^. PTP1B is a potent tyrosine phosphatase known to inactivate MET^37^, and its enzymatic function is negatively modulated by phosphorylation on several serine residues (S50, S352, and S378) in response to different stimuli^24,25^. These pieces of evidence prompted us to investigate the possible role of PTP1B in cells released from MET kinase inhibition. EBC1 and HS746T cells were treated overnight with JNJ-605 and released through a rapid washout. Concomitant with the peak of MET tyrosine re-phosphorylation, we detected a substantial increase in PTP1B serine phosphorylation on two residues (S50 and S378) both in EBC1 and in HS746T cells. The S352 was phosphorylated to a lesser extent after MET kinase inhibitor washout (Fig. 3A).

We thus verified if the AKT/mTOR axis was responsible for the phosphorylation/inactivation of PTP1B by using the publicly available TCGA phosphoproteome dataset (mTOR and PTP1B phosphorylation data were only available for breast cancer, N = 42 patients). We found that mTOR phosphorylation in patients was positively correlated with PTP1B phosphorylation, especially on S50 and S378 (https://www.cancer.gov/tcga, Fig. 3B). We, therefore, assessed the phosphorylation levels of PTP1B in EBC1 and HS746T cells after JNJ-605 washout treated with indicated inhibitors. While JNJ-605 washout provoked a substantial increase of S50 and S378, AKT and mTOR inhibition hampered PTP1B phosphorylation-dependent inactivation (Fig. 3C). These results indicate that, during inhibitor withdrawal, PTP1B is phosphorylated on S50 and S378 by the AKT/mTOR pathway and suggest that these post-translational modifications inhibit dephosphorylation of the MET receptor.

**Figure 4:**
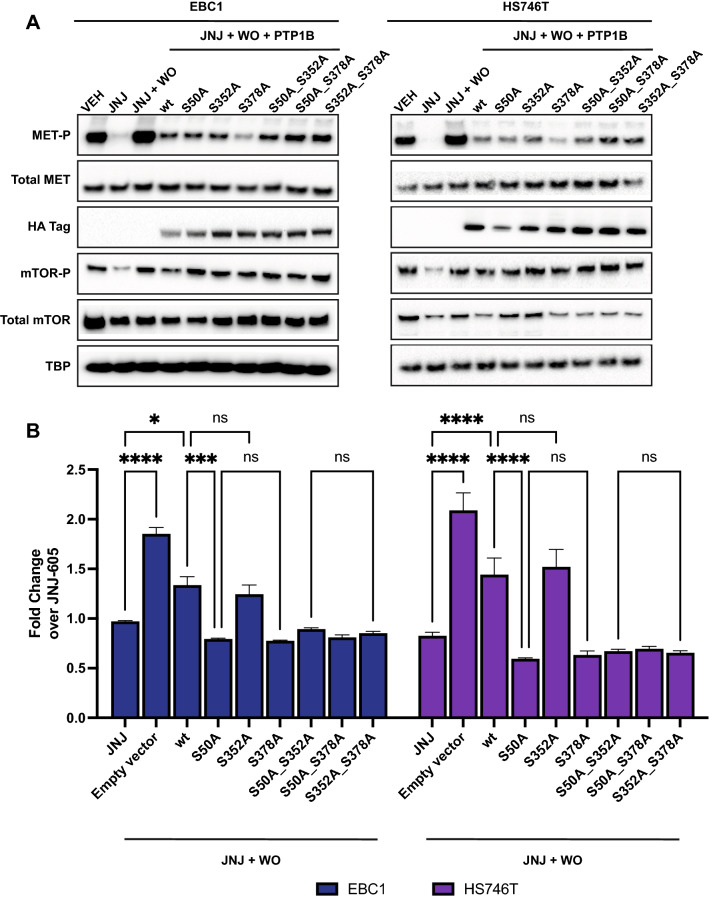
Phosphorylation on S50 and S378 inhibits the activity of PTP1B. (**A**) EBC1 and HS746T cells were treated with DMSO or JNJ-605. After WO, cells were transiently transfected with indicated vectors (JNJ + WO condition corresponds to transfection with an empty vector). All vectors containing the cDNA of *PTP1B* were N-terminally tagged with HA. Cellular lysates were immunoblotted, as indicated on the left. Black-edged rectangles delineate the blot. (**B**) Cells were treated and transfected as in (**A**) and subjected to viability assay 72 h post-WO and post-transfection. Results are the mean fold change over JNJ-605-treated cells without WO +/− SEM with N = 6. ANOVA was performed to test statistical significance with Tukey’s post-hoc test with ns: not significant; *P < 0.05; **P < 0.01; ***P < 1.10^–3^; ****P < 1.10^–4^.

We then directly tested the effect of S50 and S378 phosphorylation on the ‘flare’ effect. We generated a panel of single mutants at residues 50, 352 or 378 by replacing serine with alanine (‘phosphorylation null’ mutants, S50A, S352A, S378A). PTP1B wild type (wt) or mutants were transfected in EBC1 or HS746T cells. Overexpression of PTP1B-wt reduced phosphorylation of MET. S378A further decreased levels of MET phosphorylated (Fig. 4A). S50A mutant showed a mild effect, whereas the S352A mutant displayed no noticeable difference compared to wt PTP1B. The double mutants behaved like single mutants, suggesting that Serine 50 or Serine 378 are critically important for the ‘flare’ effect, and their influence is not additive (data not shown). Besides, overexpression of wt or mutants PTP1B did not hamper mTOR activation, further supporting that PTP1B is downstream of mTOR. Moreover, cell proliferation was hampered when PTP1B was overexpressed (Fig. 4B, wt condition). The growth was further decreased when cells were transfected with the phosphorylation-null mutants S50A or S378A. Transfection with S352A showed similar results to wt PTP1B condition (Fig. 4B). These observations support the conclusion that S50 and S378 residues of PTP1B are critical for the ‘flare’ effect.

**Figure 5:**
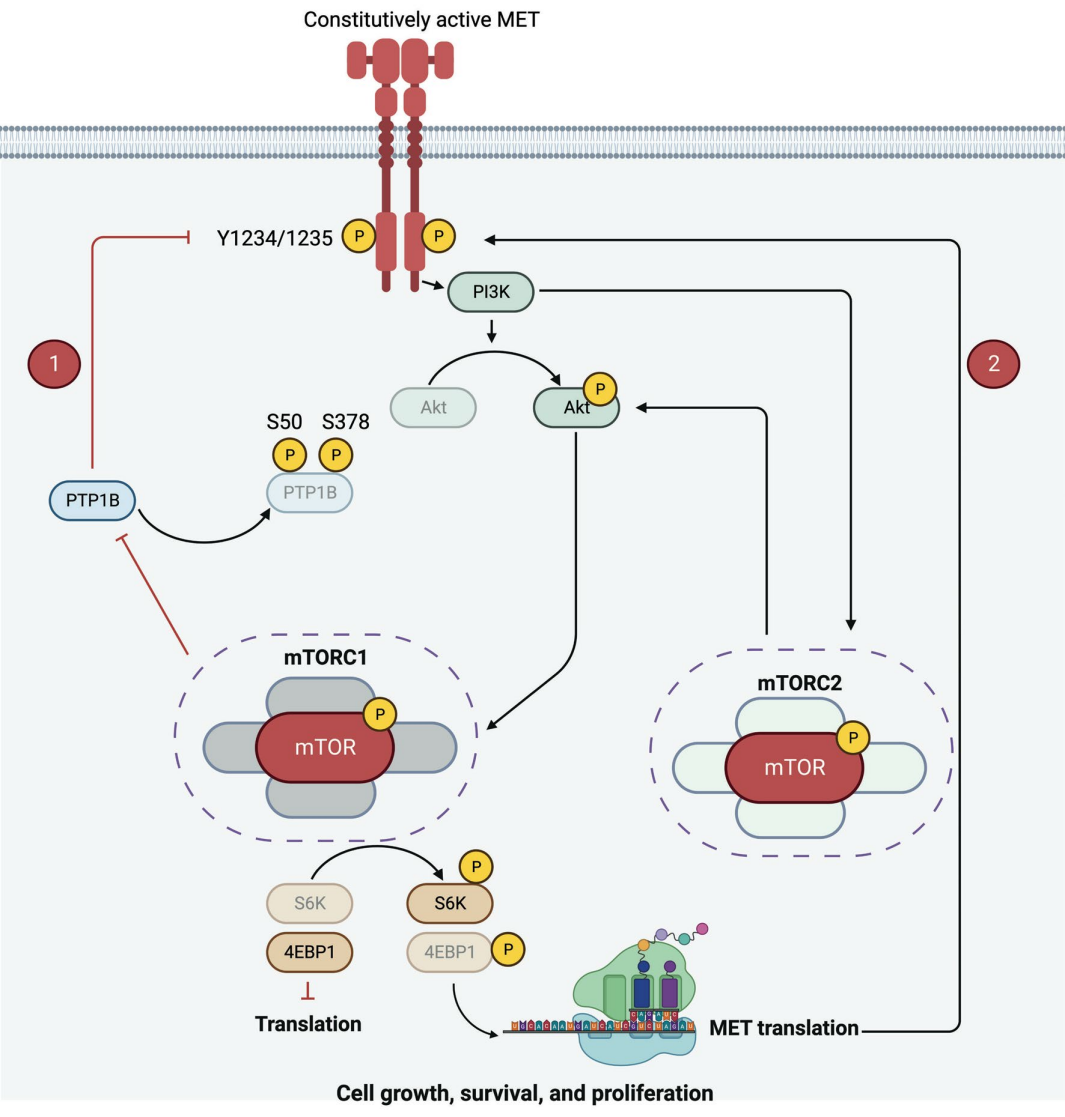
Schematics of the mechanism of the ‘flare’ effect. JNJ-605 discontinuation leads to the activation of the AKT/mTOR pathway, which drives (1) PTP1B inactivation by phosphorylation on S50 and S378, and (2) enhances translation of MET kinase. This dual feature leads to hyper-expressed and hyper-activated MET and favours the ‘flare’ effect. Created with BioRender.com.

Altogether, our work uncovers the extended mechanism of the ‘flare’ effect. After MET inhibitor withdrawal, AKT/mTOR pathway is activated, enhancing the protein synthesis of MET and inhibiting its dephosphorylation (inactivation) (schematised in Fig. 5).

## Discussion

MET phosphorylation drives its oncogenic potential^38^. We confirm that increased and sustained MET phosphorylation in cancer cells released from kinase blockade enhances proliferation^23^. These findings point to MET reactivation after inhibitor dismissal as a relevant risk of ‘rebound’ in cancer cells. This unfortunate event has been described in the clinic for EGF receptor inhibitors as the—so far unexplained—‘flare’ effect^21,22^. Like the EGF receptor, MET down-regulation is achieved by deeply intertwined processes, namely, dephosphorylation and endocytosis-mediated degradation^39^. Receptor internalisation is triggered by tyrosine auto-phosphorylation of the cytoplasmic tail. In a previous paper, we showed that inhibition of phosphorylation of MET goes along with inhibition of internalisation and accumulation at the surface^23^. Other researchers published similar observations using other small molecule inhibitors (EMD1214063, PHA665752, PF02341066)^40^. It should be noted that the receptor accumulated at the cell surface is not phosphorylated, and therefore inactive, but potentially ready to be re-activated after inhibitor withdrawal^23,40^. Here, we show that the phosphatase PTP1B is inhibited by AKT-mTOR-dependent phosphorylation upon inhibitor withdrawal. PTP1B is central to MET regulation because it dephosphorylates the activating residues Y1234/1235 on the kinase domain, inhibiting all receptor functions. PTP1B can be phosphorylated in serine, which inhibits phosphatase activity^24,25,41^. After MET kinase inhibitor withdrawal, PTP1B is inhibited by AKT-mTOR-dependent phosphorylation. PTP1B-dependent MET tyrosine dephosphorylation is therefore impaired, and MET is hyper-activated. In particular, we observed that phosphorylation of serines 50 and 378 of PTP1B play a critical role in the process, as demonstrated by the use of PTP1B non-phosphorylatable mutants. Notably, AKT and mTOR inhibition both affect phosphorylation of PTP1B and blunt reactivation of MET after JNJ-605 washout, showing that the AKT-mTOR axis is part of a MET positive feedback loop.

The AKT/MTOR pathway triggered by discontinuation of MET blockade is responsible for MET hyperphosphorylation and MET synthesis. Indeed, mTOR is a crucial regulator of translation^32^. In this article, we demonstrate that the AKT/mTOR pathway controls total MET levels. Altogether, we describe the ‘flare’ effect as the consequence of a strategy of cancer cells enhancing both MET phosphorylation (activation) and MET synthesis.

The critical role of mTOR in cancer is well-known^32,42^. This study shows its pivotal role during the ‘flare’ effect and its emergence as a promising target against tumour ‘rebound’. We demonstrate that AKT/mTOR activation follows the withdrawal of MET inhibitors, initiating a positive feedback activation of the MET pathway, responsible for the ‘flare’ effect. If these conclusions can be generalised, pulsatile treatments alternating tyrosine kinase and mTOR inhibitors could provide a promising strategy to hamper tumour growth, reducing the risk of disease ‘rebound’.

## Methods

### Plasmids and mutagenesis

The pJ3H PTP1B plasmid was a kind gift from Ben Neel (Addgene plasmid number 8601). Q5® Site-Directed mutagenesis kit (New England Biolabs®, Catalogue number E0554) was used following manufacturer’s instructions to generate Serine to Alanine mutations of PTP1B. Primers used for the mutagenesis reactions are listed in Supplementary Table 1. Plasmids used in this study are available upon request.

### Cell culture

EBC1 (Creative Bioarray, Catalogue number CSC-C6336J) and HS746T (ATCC, Catalogue number HTB-135TM) cell lines were purchased from indicated companies and were cultured respectively in RPMI and DMEM media supplemented with 10% FBS and 1% L-Glutamine. The medium was reefed every 3–4 days. All cells were regularly assessed for the absence of mycoplasma contamination. Cells were transiently transfected with Lipofectamine™ 2000 reagent following manufacturer’s instructions (ThermoFisher Scientific™, Catalogue number 1ack1668019).

### Inhibitor withdrawal

EBC1 and HS746T cells were plated at the concentration of 5 × 10^5^ cells in 6 cm dishes. Twenty-four hours later, the MET kinase inhibitor JNJ-38877605 (indicated as JNJ-605) was added at a concentration of 500 nM overnight (ON). Cells were washed three times with medium. After adding fresh medium, cells were left in culture for indicated durations. DMSO-treated cells (VEH) were processed as the JNJ-605-treated ones. When indicated, inhibitors were added to the medium immediately after the JNJ-605 washout. The different inhibitors used in this study are listed in Supplementary Table 2.

### Immunoblotting

Cells were lysed in Laemmli Lysis Buffer as previously described^31^. Cell lysates were quantified using Pierce BCA Protein Assay Kit (ThermoFisher Scientific™, Catalogue number 23225), and 15 µg of total proteins were resolved in polyacrylamide gels under denaturing conditions. After transfer, membranes (ThermoFisher Scientific™, Invitrolon™ PVDF, Catalogue number LC2005) were blotted against antibodies listed in Supplementary Table 3. Bands were revealed by chemiluminescent reaction (Merck, ECL™ Prime Western Blotting Detection Reagent, Catalogue number GERPN2232, and ECL™ Select Western Blotting Detection Reagent, Catalogue number GERPN2235).

### Immunofluorescence

Cells were plated in chamber slides (ThermoFisher Scientifi™, Nunc™ Lab-Tek™ II Chamber Slide™ System, Catalogue number 154461PK). Immunostainings were performed as previously described using antibodies listed in Supplementary Table 3. Images were acquired with the Leica TCS SP8-DLS microscope (Leica Microsystems).

### Viability assay

Cells were plated in 96-well plates at a density of 5000 cells / well. After the ON JNJ-605 treatment, cells were washed as described above and treated with the indicated inhibitors. Cell viability was assessed using CellTiter 96® Non-Radioactive Cell Proliferation Assay (Promega, Catalogue Number G4100) every day for eight days. The culture medium was renewed every three days.

### Study cohort

The publicly available TCGA dataset was used to assess PTP1B and mTOR phosphorylation levels in human cancers. On the day of analyses (June 2022), 97.3% of TCGA phosphoproteome data was based obtained from breast invasive carcinoma patients. For some patients, PTP1B and/or mTOR phosphorylation levels were unavailable and therefore not analysed. In the end, a cohort of 42 breast carcinoma patients was obtained. Patient information is available in Supplementary Table 4.

## Supplementary Information

Supplementary Information 1.

Supplementary Information 2.

## Data Availability

TCGA data were downloaded in June 2022. Data were analysed using R version 4.1.2. Raw tables and codes are available at: https://github.com/Altintas-D/Flare.git. Original western blot images are available in the Supplementary Information file.
