# Supplementary material for: An mTOR feedback loop mediates the ‘flare’ (‘rebound’) response to MET tyrosine kinase inhibition
Source: Sci Rep. 2023 Jan 25;13:1378. doi: 10.1038/s41598-023-28648-3 (PMC9876934; doi:10.1038/s41598-023-28648-3)

Original blots of Fig. 1A

MET-P

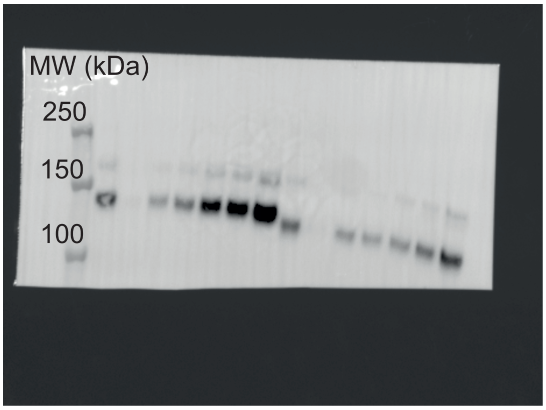

AKT-P

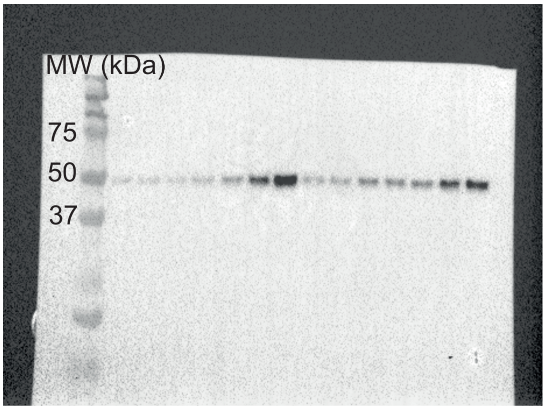

Total MET

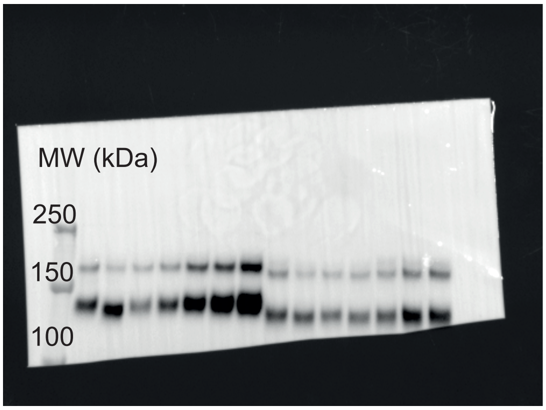

Total AKT

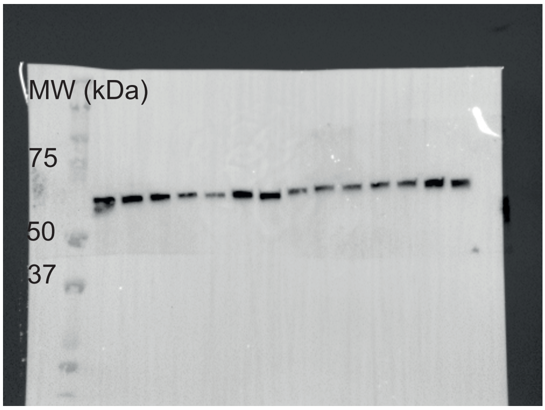

TBP

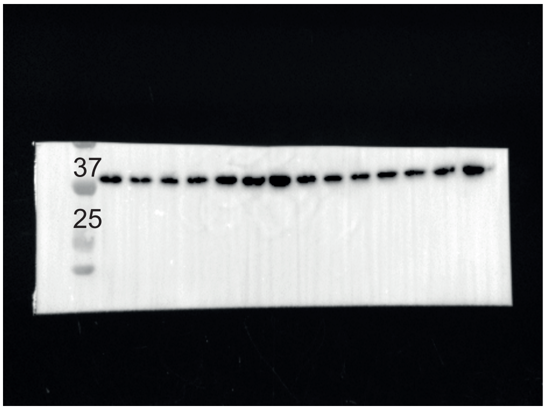

ERK1/2-P

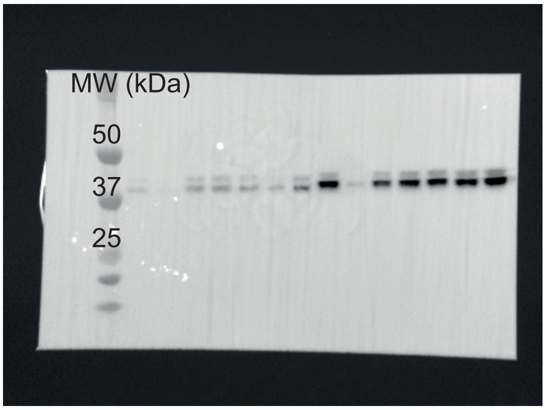

Total ERK1/2

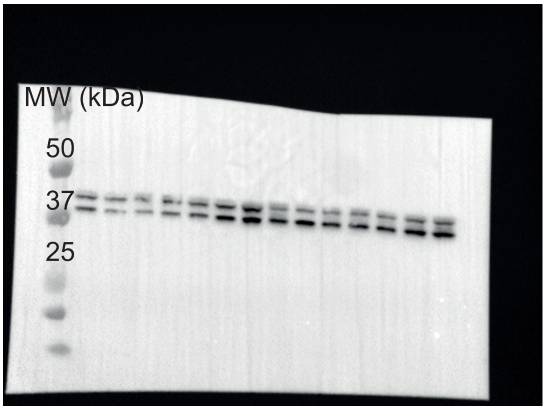

Original blots of Fig. 1C

MET-P

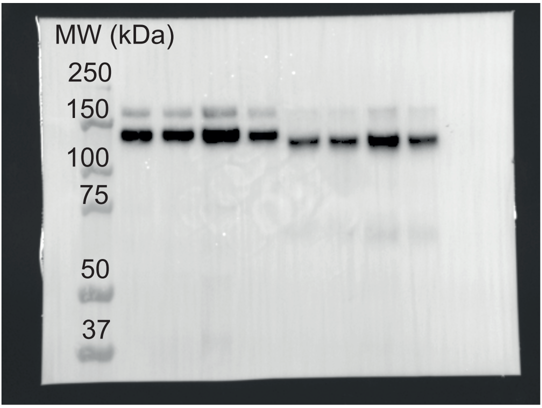

AKT-P

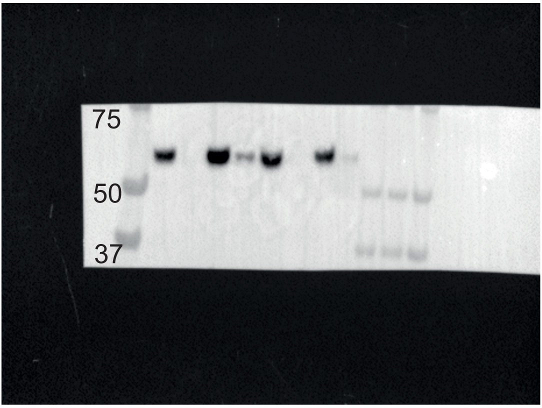

TBP

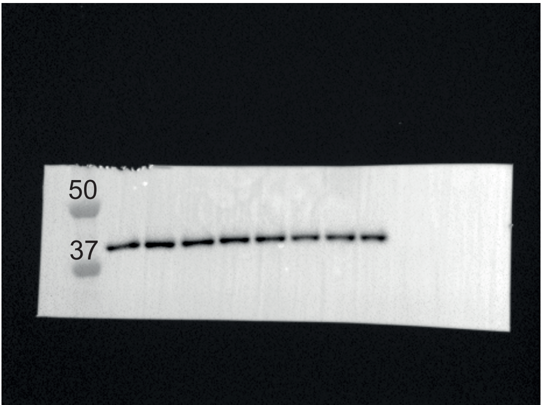

Original blots of Fig. 2A

mTOR-P

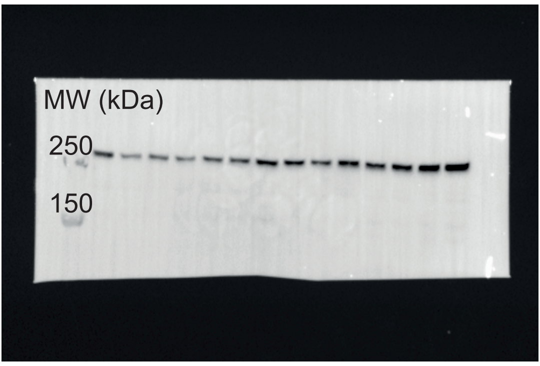

Total mTOR

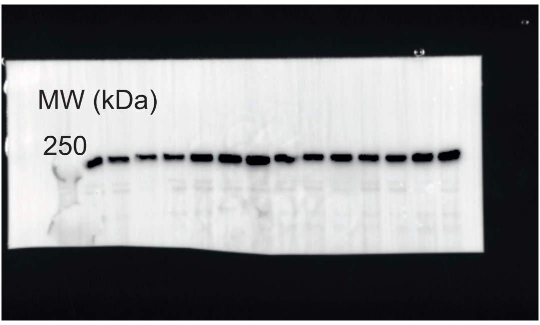

4EBP1-P

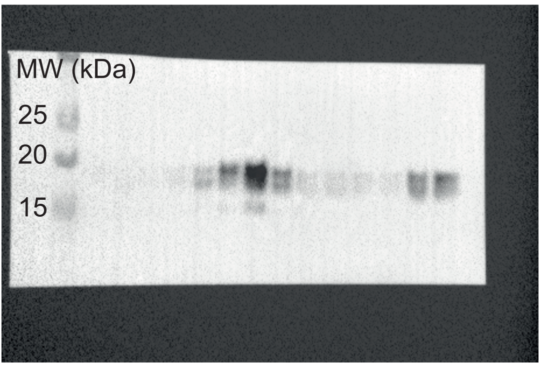

Total 4EBP1

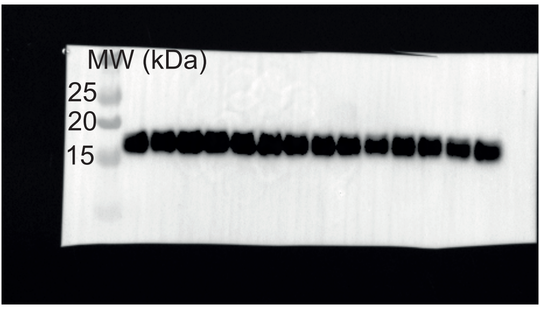

p70S6K-P

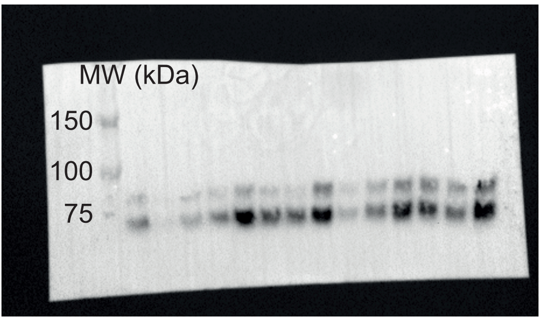

TBP

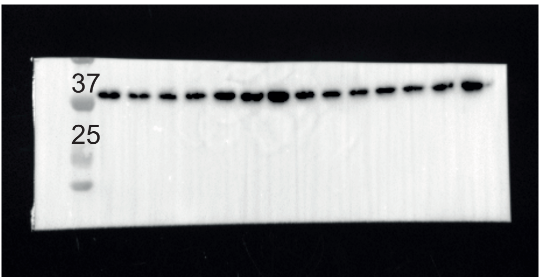

Original blots of Fig. 2B

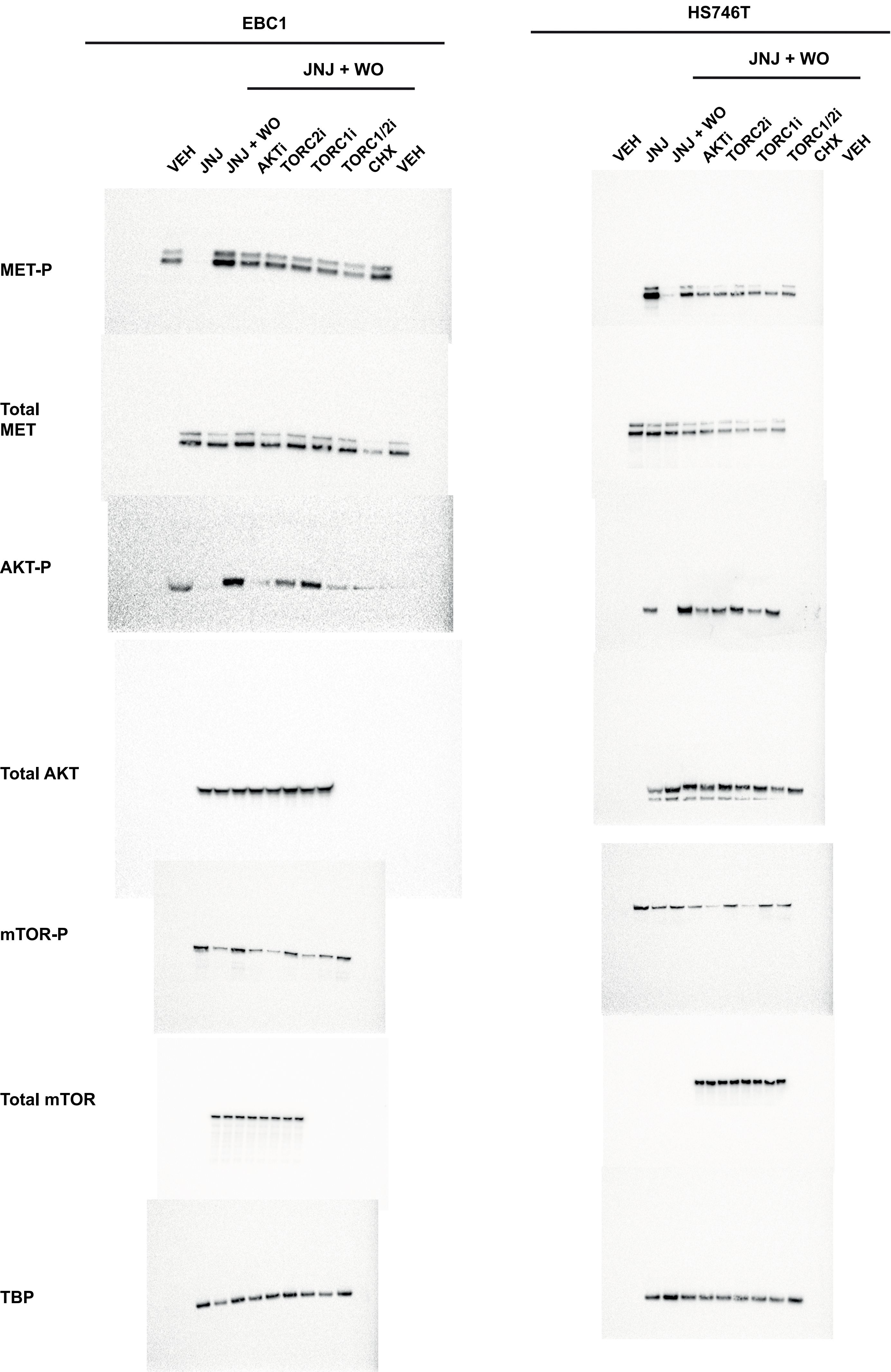

Original blots of Fig. 3A

PTP1B-P-S50

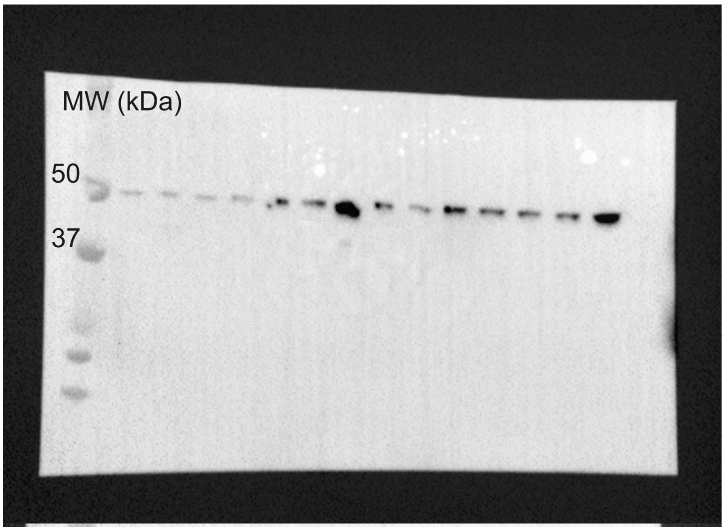

PTP1B-P-S352

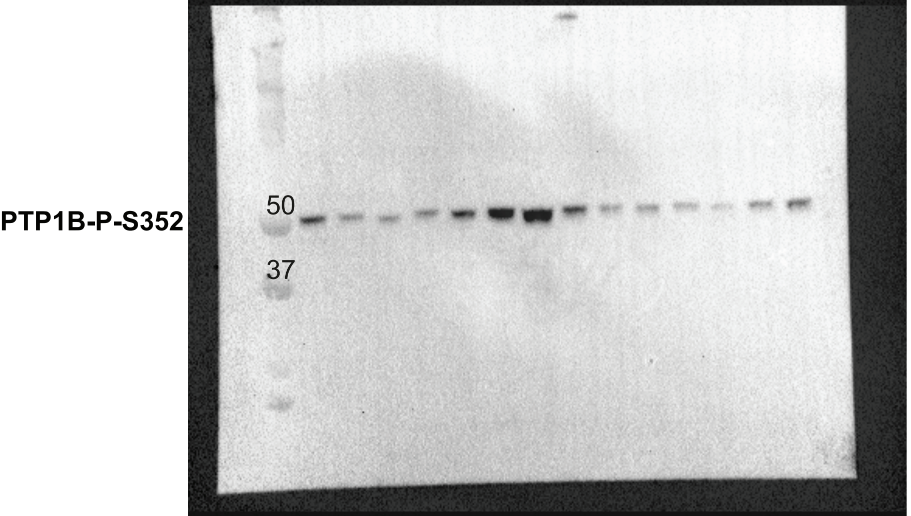

PTP1B-P-S378

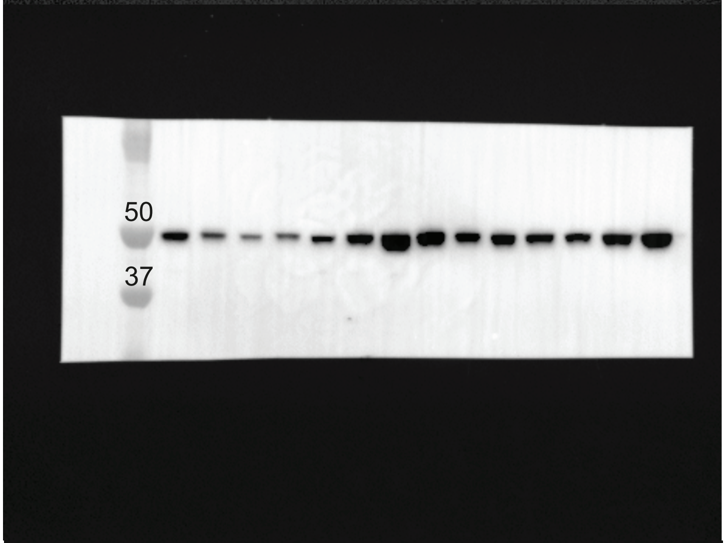

Total PTP1B

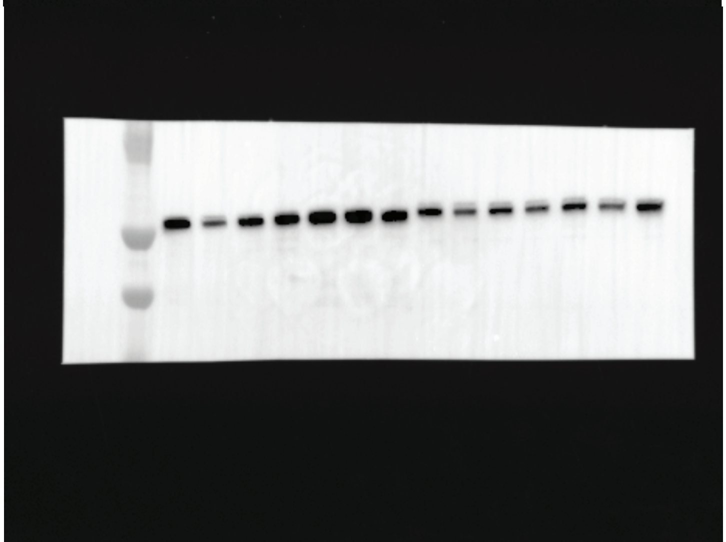

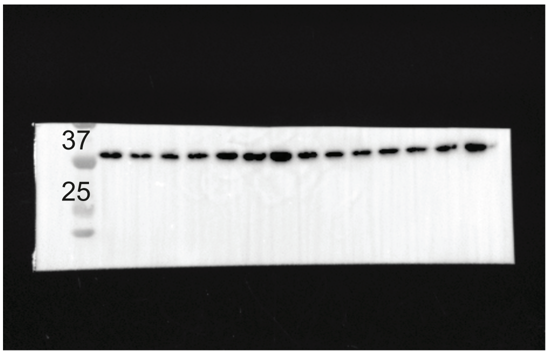

TBP

Original blots of Fig. 3C

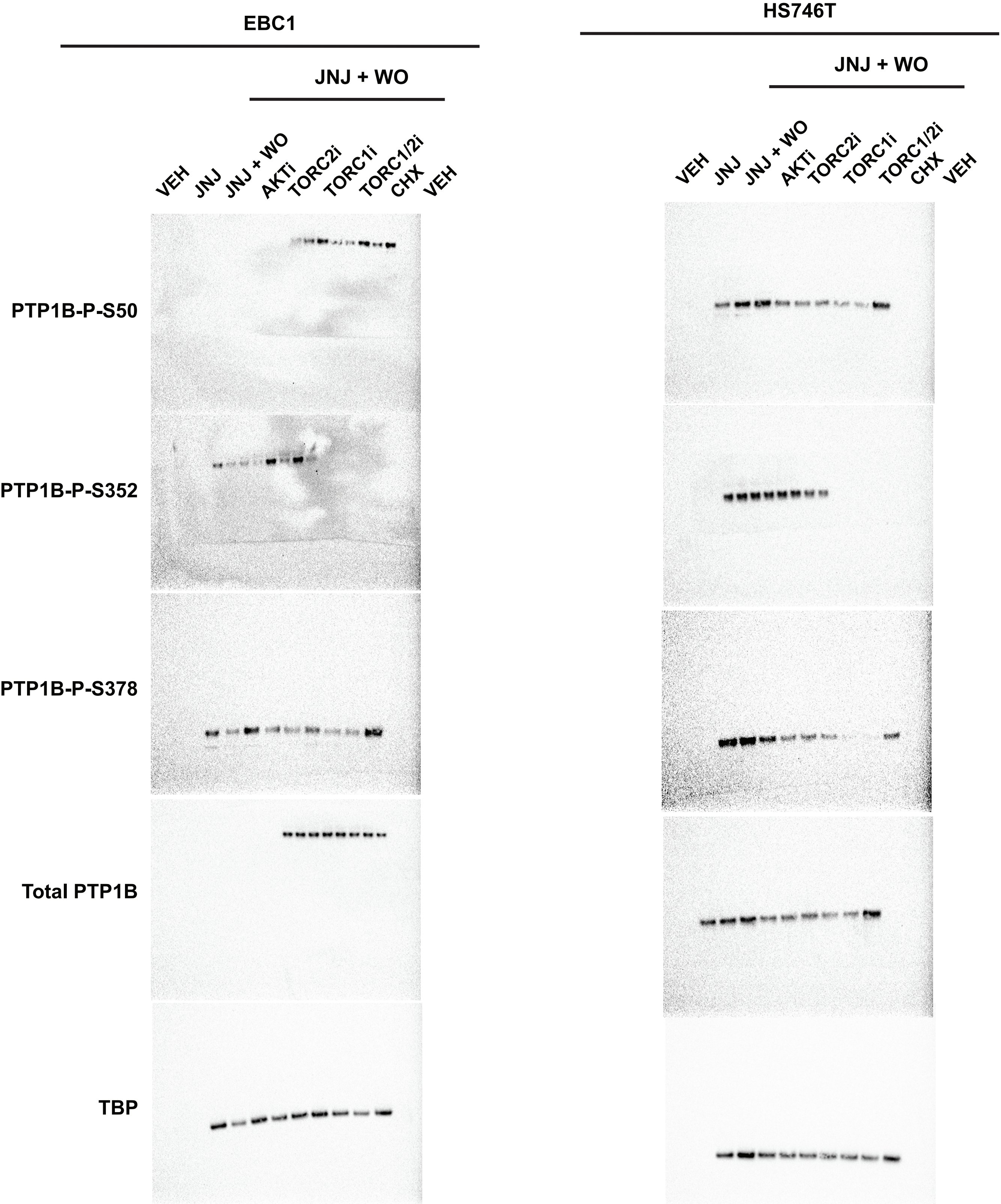

Original blots of Fig. 4A

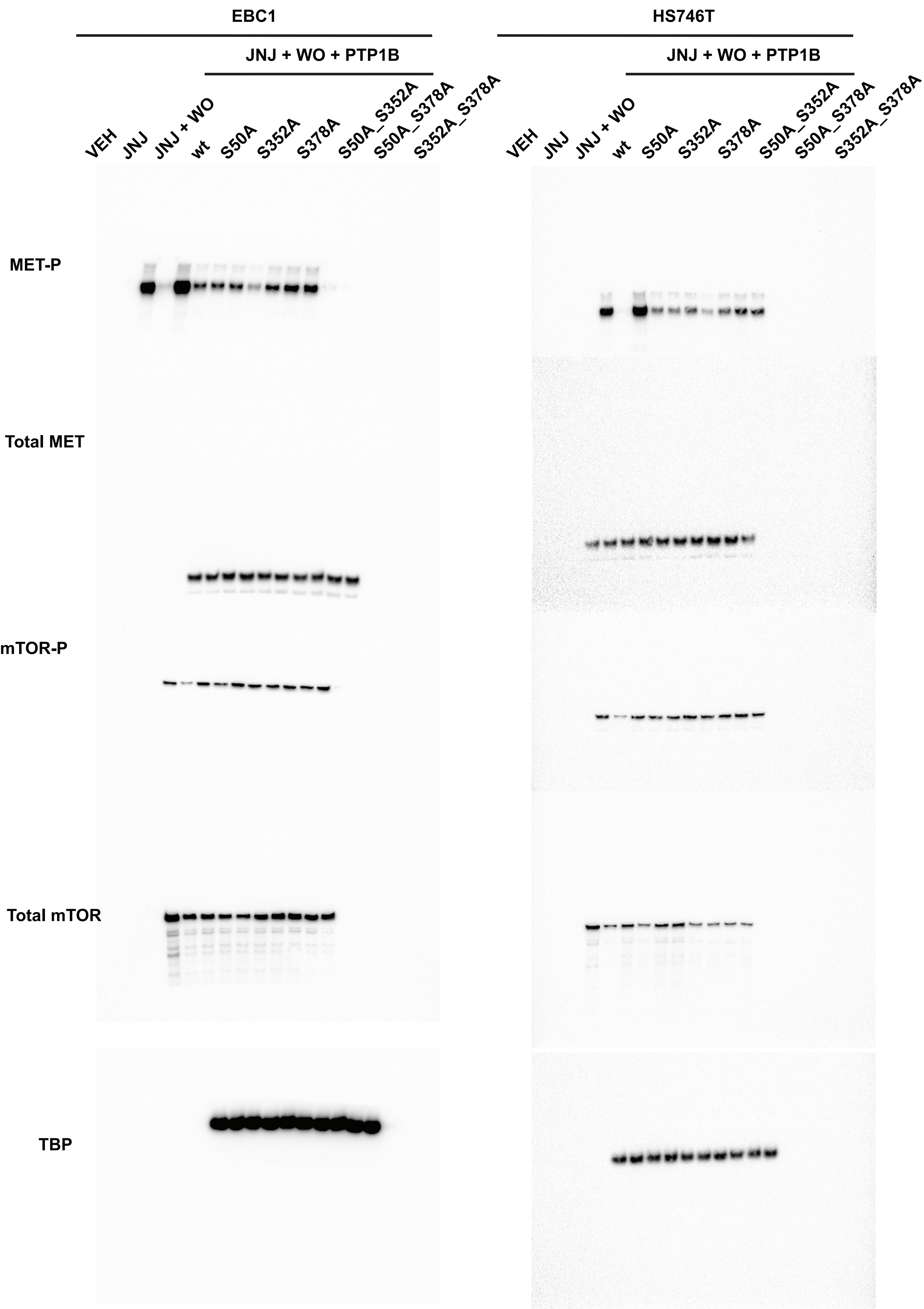

Supplement: Supplementary file 1 — Supplementary Information 1. [file 41598_2023_28648_MOESM1_ESM.pdf]
