# Supplementary material for: An mTOR feedback loop mediates the ‘flare’ (‘rebound’) response to MET tyrosine kinase inhibition
Source: Sci Rep. 2023 Jan 25;13:1378. doi: 10.1038/s41598-023-28648-3 (PMC9876934; doi:10.1038/s41598-023-28648-3)
Supplement: Supplementary file 2 — Supplementary Information 2. [file 41598_2023_28648_MOESM2_ESM.docx]

| Name | Sequence | Tm | Description |
| --- | --- | --- | --- |
| S50A_F | CAGAGACGTCgctCCCTTTGACCATAG | 58 | PTP1B S50A mutation |
| S50A_R | TACCTATTTCGGTTTTTGTTC | 58 | PTP1B S50A mutation |
| S352A_F | AGAAAAAGGAgccCCCTTAAATGCCGC | 63 | PTP1B S352A mutation |
| S352A_R | TCCTTGATGGGGCAGTCT | 63 | PTP1B S352A mutation |
| S378A_F | CGTGGGGGGAgccCTTCGAGGTG | 62 | PTP1B S378A mutation |
| S378A_R | ACCCGACTTCTAACTTCAG | 62 | PTP1B S378A mutation |

Supplementary Table 1: Primers used for site-directed mutagenesis. PTP1B cDNA sequence was mutated using the Q5® Site-Directed Mutagenesis Kit (New England Biolabs, catalog #E0554) following the manufacturer’s instructions to generate pJ3H PTP1B S50A, pJ3H PTP1B S352A, pJ3H PTP1B S378A, pJ3H PTP1B S50A_S352A, pJ3H PTP1B S50A_S378A, and pJ3H PTP1B S352A_S378A plasmids.

| Compound | Company | Code | Solvant | Treatment dose (uM) | Treatment duration | Targets |
| --- | --- | --- | --- | --- | --- | --- |
| JA-AB2-011 | MedChemExpress | HY-122022 | DMSO | 1 | 1-3days | mTORC2 |
| Rapamycin | MedChemExpress | HY-10219 | DMSO | 0,1 | 1-3days | mTORC1 |
| Temsirolimus | MedChemExpress | HY-50910 | DMSO | 1 | 1-3days | mTORC1 and 2 |
| JNJ-38877605 | Selleckchem | S1114 | DMSO | 0,5 | 1-3days | MET |
| MK-2206 | Selleckchem | S1078 | DMSO | 1 | 1-3days | AKT |
| Cycloheximide | ThermoFisher | Cat 357420010 | Ethanol | 25 | 1-3days | Translation |

Supplementary Table 2: Inhibitors used in the study.

| Name | Reference | Company | Species | Size (kDa) | Dilution |
| --- | --- | --- | --- | --- | --- |
| P-MET-Y1234/1235 | 3077 | Cell Signalling | Rabbit | 145 | 1/500 WB, 1/200 IF |
| Total MET | 3148 | Cell Signalling | Mouse | 145 | 1/500 WB |
| Total MET | 18-7366 | ThermoFisher | Mouse | 145 | 1/200 IF |
| Phospho-Akt (Ser473) Antibody | 9271 | Cell Signalling | Rabbit | 60 | 1/1000 WB |
| Total AKT | 9272 | Cell Signalling | Rabbit | 60 | 1/1000 WB |
| P-mTOR-S2448 | 5536 | Cell Signalling | Rabbit | 289 | 1/1000 WB |
| Total mTOR | 2983 | Cell Signalling | Rabbit | 289 | 1/1000 WB |
| P-Rictor-T1135 | 3806 | Cell Signalling | Rabbit | 200 | 1/1000 WB |
| Total Rictor | 2114 | Cell Signalling | Rabbit | 200 | 1/1000 WB |
| PTP1B-P-S50 | ab62320 | Abcam | Rabbit | 50 | 1/1000 WB |
| PTP1B-P-S378 | ab76239 | Abcam | Rabbit | 50 | 1/1000 WB |
| PTP1B-P-S352 | ab62330 | Abcam | Rabbit | 50 | 1/2000 WB |
| Total PTP1B | 5311 | Cell Signalling | Rabbit | 50 | 1/1000 WB |
| P-p70S6K-T389 | 9205 | Cell Signalling | Rabbit | 70 | 1/1000 WB |
| Phospho-p44/42 MAPK (Erk1/2) (Thr202/Tyr204) Antibody | 9101 | Cell Signalling | Rabbit | 42, 44 | 1/5000 WB |
| p44/42 MAPK (Erk1/2) Antibody | 9102 | Cell Signalling | Rabbit | 42, 44 | 1/5000 WB |
| HA Tag | 2367 | Cell Signalling | Mouse | NA | 1/1000 WB |
| Total 4EBP1 | 9644 | Cell Signalling | Rabbit | 15-20 | 1/1000 WB |
| e | 2855 | Cell Signalling | Rabbit | 15-20 | 1/1000 WB |

Supplementary Table 3: Antibodies used in the study. WB: western blot; IF: immunofluorescence

| Sample ID | Disease | Sex | Age at Diagnosis |
| --- | --- | --- | --- |
| TCGA-A2-A0D2-01 | Breast Cancer (Breast Invasive Ductal Carcinoma) | Female | 45 |
| TCGA-A2-A0EQ-01 | Breast Cancer (Breast Invasive Ductal Carcinoma) | Female | 64 |
| TCGA-A2-A0EV-01 | Breast Cancer (Breast Invasive Ductal Carcinoma) | Female | 80 |
| TCGA-A2-A0EY-01 | Breast Cancer (Breast Invasive Ductal Carcinoma) | Female | 62 |
| TCGA-A2-A0T6-01 | Breast Cancer (Breast Invasive Lobular Carcinoma) | Female | 50 |
| TCGA-A2-A0T7-01 | Breast Cancer (Breast Invasive Ductal Carcinoma) | Female | 51 |
| TCGA-A2-A0YF-01 | Breast Cancer (Breast Invasive Ductal Carcinoma) | Female | 67 |
| TCGA-A2-A0YI-01 | Breast Cancer (Breast Invasive Ductal Carcinoma) | Female | 62 |
| TCGA-A7-A0CD-01 | Breast Cancer (Breast Invasive Ductal Carcinoma) | Female | 66 |
| TCGA-A8-A079-01 | Breast Cancer (Breast Invasive Ductal Carcinoma) | Female | 69 |
| TCGA-A8-A08G-01 | Breast Cancer (Breast Invasive Ductal Carcinoma) | Female | 41 |
| TCGA-A8-A09G-01 | Breast Cancer (Breast Invasive Ductal Carcinoma) | Female | 79 |
| TCGA-AN-A04A-01 | Breast Cancer (Breast Invasive Ductal Carcinoma) | Female | 36 |
| TCGA-AN-A0AL-01 | Breast Cancer (Breast Invasive Ductal Carcinoma) | Female | 41 |
| TCGA-AN-A0AM-01 | Breast Cancer (Breast Invasive Ductal Carcinoma) | Female | 56 |
| TCGA-AN-A0AS-01 | Breast Cancer (Breast Invasive Ductal Carcinoma) | Female | 70 |
| TCGA-AN-A0FL-01 | Breast Cancer (Breast Invasive Ductal Carcinoma) | Female | 62 |
| TCGA-AO-A0JJ-01 | Breast Cancer (Breast Invasive Lobular Carcinoma) | Female | 54 |
| TCGA-AO-A0JM-01 | Breast Cancer (Breast Invasive Ductal Carcinoma) | Female | 40 |
| TCGA-AO-A12D-01 | Breast Cancer (Breast Invasive Ductal Carcinoma) | Female | 43 |
| TCGA-AR-A0TV-01 | Breast Cancer (Breast Invasive Ductal Carcinoma) | Female | 66 |
| TCGA-AR-A1AS-01 | Breast Cancer (Breast Invasive Ductal Carcinoma) | Female | 54 |
| TCGA-AR-A1AV-01 | Breast Cancer (Breast Invasive Ductal Carcinoma) | Male | 68 |
| TCGA-AR-A1AW-01 | Breast Cancer (Breast Invasive Ductal Carcinoma) | Female | 65 |
| TCGA-BH-A0AV-01 | Breast Cancer (Breast Invasive Ductal Carcinoma) | Female | 52 |
| TCGA-BH-A0C1-01 | Breast Cancer (Breast Invasive Lobular Carcinoma) | Female | 61 |
| TCGA-BH-A0DD-01 | Breast Cancer (Breast Invasive Ductal Carcinoma) | Male | 58 |
| TCGA-BH-A0DG-01 | Breast Cancer (Breast Invasive Ductal Carcinoma) | Female | 30 |
| TCGA-BH-A0E9-01 | Breast Cancer (Breast Invasive Lobular Carcinoma) | Female | 53 |
| TCGA-BH-A18U-01 | Breast Cancer (Breast Invasive Ductal Carcinoma) | Female | 72 |
| TCGA-C8-A12P-01 | Breast Cancer (Breast Invasive Ductal Carcinoma) | Female | 55 |
| TCGA-C8-A12Q-01 | Breast Cancer (Breast Invasive Ductal Carcinoma) | Female | 78 |
| TCGA-C8-A12U-01 | Breast Cancer (Breast Invasive Ductal Carcinoma) | Female | 46 |
| TCGA-C8-A12V-01 | Breast Cancer (Breast Invasive Ductal Carcinoma) | Female | 55 |
| TCGA-C8-A12W-01 | Breast Cancer (Breast Invasive Ductal Carcinoma) | Female | 49 |
| TCGA-C8-A12Z-01 | Breast Cancer (Breast Invasive Ductal Carcinoma) | Female | 45 |
| TCGA-C8-A131-01 | Breast Cancer (Breast Invasive Ductal Carcinoma) | Female | 82 |
| TCGA-C8-A134-01 | Breast Cancer (Breast Invasive Ductal Carcinoma) | Female | 52 |
| TCGA-C8-A135-01 | Breast Cancer (Breast Invasive Ductal Carcinoma) | Female | 64 |
| TCGA-D8-A142-01 | Breast Cancer (Breast Invasive Ductal Carcinoma) | Female | 74 |
| TCGA-E2-A158-01 | Breast Cancer (Breast Invasive Ductal Carcinoma) | Female | 43 |
| TCGA-E2-A15A-01 | Breast Cancer (Breast Invasive Ductal Carcinoma) | Female | 45 |

Supplementary Table 4: Patient information of the TCGA phosphoproteome dataset used in this study.
